# American Academy of Dental Science

**Published:** 1876-02

**Authors:** Geo. T. Moffatt

**Affiliations:** Secretary of the Committee, No. 1 Hotel Boylsten, Boston, Mass.


### ARTICLE V.

*To Editor of American Journal of Dental Science:*

DEAR SIR—

Will you kindly give place to the following announcement in the February number of the "JOURNAL OF DENTAL SCIENCE," and oblige

Yours very truly,

GEO. T. MOFFATT.

---

At the December monthly meeting of the "*American Academy of Dental Science*," Dr. D. M. Parker, member of the Centennial Board of State Managers, and President of the Academy, in the chair, it was voted unanimously that the Academy shall endeavor to present in some suitable manner the claims of Dentistry, or more properly, of "Oral Science," at the coming "Centennial" at Philadelphia.

A committee of ten was appointed to make the necessary arrangements, and they have held weekly meetings from that time to the present. To this committee have been added as "advisory members," representative men throughout the country.

In order to still further aid the committee in this undertaking, they now request the co-operation of all members of the profession who may be interested in the movement, and would solicit any information pertinent to the subject that any one may have in their power to give. They particularly desire *facts* relative to the *early history* of the profession in this country; as to individual practitioners and the efforts that have been made at securing a suitable and thorough education in this *specialty of medicine.*

All information bearing upon the above points, will be gratefully received by the committee, and may be sent to

GEO. T. MOFFATT, M. D.,

*Secretary of the Committee,*

No. 1 Hotel Boylsten,

*Boston, Mass.*

